# Composition, training needs and independence of ethics review committees across Africa: are the gate-keepers rising to the emerging challenges?

**DOI:** 10.1136/jme.2008.025189

**Published:** 2009-02-20

**Authors:** A Nyika, W Kilama, R Chilengi, G Tangwa, P Tindana, P Ndebele, J Ikingura

**Affiliations:** 1 African Malaria Network Trust (AMANET), Dar es Salaam, Tanzania; 2 University of Yaoundé 1, Yaoundé, Cameroon; 3 Navrongo Health Research Centre, Navrongo, Ghana; 4 College of Medicine, University of Malawi, Blantyre, Malawi; 5 National Institute for Medical Research (NIMR), Dar es Salaam, Tanzania

## Abstract

**Background:**

The high disease burden of Africa, the emergence of new diseases and efforts to address the 10/90 gap have led to an unprecedented increase in health research activities in Africa. Consequently, there is an increase in the volume and complexity of protocols that ethics review committees in Africa have to review.

**Methods:**

With a grant from the Bill and Melinda Gates Foundation, the African Malaria Network Trust (AMANET) undertook a survey of 31 ethics review committees (ERCs) across sub-Saharan Africa as an initial step to a comprehensive capacity-strengthening programme. The number of members per committee ranged from 3 to 21, with an average of 11. Members of 10 institutional committees were all from the institution where the committees were based, raising prima facie questions as to whether independence and objectivity could be guaranteed in the review work of such committees.

**Results:**

The majority of the committees (92%) cited scientific design of clinical trials as the area needing the most attention in terms of training, followed by determination of risks and benefits and monitoring of research. The survey showed that 38% of the ERC members did not receive any form of training. In the light of the increasing complexity and numbers of health research studies being conducted in Africa, this deficit requires immediate attention.

**Outcome:**

The survey identified areas of weakness in the operations of ERCs in Africa. Consequently, AMANET is addressing the identified needs and weaknesses through a 4-year capacity-building project.

Recent concerted efforts to address the Grand Challenges in Global Health^1^ ^2^ and the 10/90 gap^3^ and to achieve the United Nations Millennium Development Goals (http://www.un.org/millenniumgoals/) have contributed to an unprecedented increase in health research involving humans in Africa. In the wake of such an increase in health research on mostly poverty-stricken and poorly educated populations, given Africa’s weak civic protection systems, it is imperative that attention be paid to the ethical review capacity of African health institutions. Review of research protocols before implementation is now regarded as one of the cornerstones of ethical research involving human participants, and some countries have made it a legal requirement.^4^ Various international and national guidelines also stipulate that ethical approval be a prerequisite for the commencement of research involving humans.

The main purpose of reviewing research protocols is to ensure that the research meets internationally acceptable scientific and ethical standards. It would be unethical for poorly designed research involving human beings to be approved, since data generated from such research would not contribute to the improvement of disease prevention or management. A holistic approach to reviewing research is critical, since issues that relate to ethical principles of autonomy, beneficence, non-maleficence and justice are equally important. One approach that has been proposed looks at seven requirements that should be considered when reviewing protocols, namely, the value of the research in terms of potential to improve health and/or knowledge, scientific validity in terms of experimental design, fair selection of participants in light of the scientific objectives of the research, favourable risk:benefit ratio with potential benefits outweighing potential risks, independent ethical review of the research before implementation, informed consent that emphasises voluntary participation, and respect for the participants recruited.^5^ In addition, community engagement has recently been recognised as a critical activity that helps to create an amicable relationship between researchers and the communities from which participants are drawn and demonstrates respect for communities as partners in research.^6^ ^7^ It is imperative that ethical review committees (ERCs) that review the protocols are adequately knowledgeable about all these requirements; otherwise the welfare of people is compromised by approval of unethical research or wrongful rejection of scientifically and ethically sound research.

Although the requirements could be assessed during the review process, implementation of approved research protocols in the field, especially in developing countries, is bound to encounter practical challenges that are attributable to socio-economic factors.^8^^–^13 Thus, ethical approval alone does not necessarily ensure protection of the safety and welfare of research participants throughout the research; hence the need for approved research to be monitored by ERCs. Ethical review and subsequent monitoring of health research require adequate resources and trained ERCs, which are limited in various ways in most African committees.

Thus, although the majority of countries in Africa are reported to now have at least some form of ethical review process in place,^14^^–^16 the operations of these processes are generally hindered by a combination of challenges, including scarcity of resources, inadequate training of members and poor staffing levels.^14^ ^16^ ^17^ For instance, a study on health research ethics review and needs of institutional ERCs in Tanzania showed that 49% of 45 respondents had not had any training in health research ethics review.^18^ Milford and colleagues also reported on the extent to which limited resources available to ethics committees in Africa could affect preparations for HIV vaccine trials.^17^ Another example is a case study of 12 African ERCs that showed inadequate training of committee members and shortage of resources to be some of the major challenges faced by the committees.^19^

In light of the relatively weak ethical review capacity in Africa, it is encouraging to note that a number of not-for-profit African organisations are involved in capacity-building programmes. The South African Research Ethics Training Initiative (SARETI) (http://shsph.up.ac.za/sareti/sareti.htm), which is based at the universities of KwaZulu-Natal and Pretoria in South Africa, provides training in ethics to African researchers and ERC members through short-term fellowships and long-term educational programmes. Another programme based in South Africa is the International Research Ethics Network for Southern Africa (IRENSA) (http://www.irensa.org), based at the University of Cape Town, which runs short-term training programmes for mid-career African academics, scientists, clinicians and members of ERCs who generally cannot enrol for long-term, full-time programmes. An additional organisation involved in providing educational programmes in Africa is the Training and Resources in Research Ethics Evaluation (TRREE) (http://www.trree.org/site/en_home.phtml) for Africa, which focuses on development of research ethics educational programmes for e-learning and provision of e-resources.

The African Malaria Network Trust (AMANET) (http://www.amanet-trust.org) is also a not-for-profit organisation that was formed in 2002, succeeding the then African Malaria Vaccine Testing Network, founded in 1995 to promote malaria vaccine trials in Africa. Although the broad objective of AMANET is still the same as that of its predecessor, the roles and activities of AMANET have been expanded to include (1) trial site development for malaria vaccine trials, which entails infrastructural development and training of research personnel in various scientific fields, (2) strengthening of ethical review capacity in Africa and (3) sponsorship of malaria vaccine clinical trials.

The current study by AMANET was aimed at finding specific gaps in the ethical review process in Africa with a view to effectively implementing a capacity-building programme tailor-made for the identified needs. Having such empirical data would go a long way towards ensuring that any interventions would complement efforts by others in this field rather than duplicate their activities, although a certain amount of overlap is both inevitable and harmless. The paucity of resources for ethical review process in Africa and the need to strengthen the process through various training programmes make it critical for the organisations working in this field to streamline their activities and programmes and promote synergistic collaborative efforts. Indeed, the efforts of the organisations are beginning to bear fruit. For instance, two ethicists working in the AMANET programmes are products of the SARETI training programmes, and an additional ethicist working with AMANET also actively takes part in both IRENSA and SARETI programmes.

In order to fill the gaps identified by the needs assessment survey, AMANET targeted ERCs as entities rather than individual members of the committees, an aspect being addressed by other organisations. Collaboration with the Pan African Bioethics Initiative started at the design stage of the current project and has continued to the implementation stage, where the two organisations jointly conduct some training workshops on health research ethics. This paper reports on the findings of the needs assessment survey and gives an overview of a longitudinal capacity-building project by AMANET.

## METHODS

Information on the capacities and needs of ERCs in Africa was obtained through presentations by selected ethics committee members during three workshops organised by AMANET in Dar es Salaam and in Addis Ababa and a needs assessment survey conducted across Africa. The data gathered through the two methods were pooled together for analyses.

### Ethics committee presentations at AMANET health research ethics workshops

Members of ethics committees attending the AMANET series of health research ethics workshops during 2007 were requested to present to the workshop participants the status and operations of their committees. The participants were requested to prepare presentations covering such issues as the committees’ composition, standard operating procedures, sources of funding, type of data management and archiving system, ethical review process and workload. This allowed interactive discussions on the shortcomings, strengths and needs of the various committees.

### Needs assessment survey

A survey questionnaire, containing 103 questions covering general identifying information and sections on the establishment of the ERCs, membership and professional background of members, funding of the committees and ethical review process for 2005 and 2006 was designed and pilot tested at institutions in southern Africa, East Africa and West Africa. A French version of the questionnaire was prepared for use in francophone countries. Six surveyors all qualified in health research ethics were appointed to conduct physical surveys across sub-Saharan Africa. The respondents were either the chairpersons or administrators of the ethics committees surveyed. The completed questionnaires were compiled and double-entered in Microsoft Excel and analysed in Stata version 10 (Stata Corporation, College Station, Texas, USA) by a statistician.

## RESULTS

### Coverage

A response rate of about 84% (31/37) was achieved, making this the most comprehensive survey of ERCs in Africa that the authors are aware of. A total of 12 institutional ERCs from nine African countries gave presentations at two AMANET health research ethics training workshops held in Dar es Salaam, Tanzania (May and August 2007), and a third workshop held in Addis Ababa, Ethiopia, in September 2007. Gaps and shortcomings of the ERCs were identified and possible solutions explored during interactive discussions that followed each presentation. The presentations covered Cameroon, Ethiopia, Ghana, Kenya, Mali, Malawi, Nigeria, Senegal, Tanzania, Gambia, Uganda and Zambia. The ERCs that presented were among the 31 respondents that were interviewed by surveyors. The countries covered in the survey, which include anglophone, francophone and lusophone countries, are shown in table 1.

**Table 1:** Number and nature of ethics review committees (ERCs) surveyed per country

Country	Number of ERCs	Affiliation
Burkina Faso	2	All research-institute based
Cameroon	2	All research-institute based
Ethiopia	2	All research-institute based
Gabon	1	Research-institute based
Gambia	1	National
Ghana	5	4 research-institute based, 1 university based
Kenya	1	National
Malawi	1	University based
Mali	1	University based
Mozambique	1	National
Nigeria	4	1 university based, 3 research-institute based
Rwanda	1	National
Senegal	1	National
Sudan	1	University based
Tanzania	4	1 national, 2 university based, 1 research-institute based
Uganda	1	University based
Zambia	1	Research-institute based
Zimbabwe	1	National
Total	31	

### Composition of the ERCs

**Figure 1:**
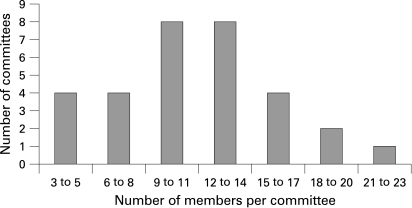
Composition of ethics review committees.

As shown in fig 1, the ERCs ranged in size from three to 21 members. The 31 ERCs surveyed had a total of 345 members, the average number of members per committee being 11. Regarding gender balance, about 33% (112/345) of the members of all 31 committees were female. Ten committees (32%) did not have any external members (that is, members not affiliated to the parent institutions where the respective committees were based). As shown in table 1, a total of seven committees are national, while seven and 17 are affiliated to universities and health research institutes, respectively.

### Constraints reported by respondents

The top five training needs cited by the respondents were scientific design of clinical trials, risk assessment of clinical trials, understanding of trial phases, monitoring of approved studies, and handling of issues surrounding post-trial access to benefits. Table 2 shows the major constraints that were highlighted by the respondents, the top two being inadequacy of resources available to the committees and limited expertise in the committees to review complex studies. Other frequently mentioned constraints were pressure from researchers, weak participation of members and low rating of the importance of the functions of ERCs. None of the respondents had electronic data management and archiving systems in place; they all relied on cumbersome, paper-based systems.

**Table 2:** Constraints hindering operations of ethics review committees (ERCs)

Constraints	Number of ERCs*
Insufficiency of resources	25/30
Lack of/insufficient expertise on ethical review	13/30
Pressure from researchers	11/30
Lack of active/consistent participation of members	11/30
Lack of recognition of the importance of ERC functions	11/30
None or poorly supported by the institute	10/30
Not completely independent	4/30
Pressure from sponsors	3/30
Unequal treatment of applicants in review	1/27
Biased committee members	0/27

*Respondents skipped some questions.

### Duration of training, and training needs of ERCs

Overall, 38% of the members had not undergone any form of training in health research ethics. Table 3 shows the duration of training courses attended by the members of the surveyed committees.

**Table 3:** Duration of training of committee members reported by respondents

Duration of training	Number of committee members
None	132
1 day	13
2–3 days	42
4–7 days	92
>7 days	48
Online training	14
Not known	4
Total	345

Table 4 shows the training needs of ethics committees. Topics related to understanding the scientific design of research protocols in intervention trials were reported as being the greatest need, while topics related to identification of appropriate subjects scored the least. Other important topics identified include determination of potential risks of vaccines, trial phase determinants and monitoring/oversight activities.

**Table 4:** Training needs of ERCs in Africa, ranked by respondents

Training needs	Ranking				
	Very important	Quite important	Important	Not important	Total institutions*
Scientific design issues in intervention trials	27		2		29
Determination of potential risks of malaria vaccine research	25	3		1	29
Determinations to run phases (I,II,III) in a country or community	25	2	1		28
Monitoring and oversight	23	5	1		29
Post-trial access to benefits (eg, successful intervention)	20	6	1	1	28
The interpretation of preclinical studies	19	7	3		29
The use of placebo in controlled trials	16	9	3	1	29
Assessment of understanding for informed consent	16	10	1	1	28
Assessment of anticipated benefits	15	8	5	1	29
Assessment of cultural sensitivity for informed consent	15	9	3	1	28
Community participation	14	11	3	1	29
Determination of appropriate subject selection in vulnerable population	14	8	5	1	28
Determination of appropriate subject selection with regard to women	13	6	8	1	28
Incentives for participation	12	10	5	1	28
Social and behavioural studies	12	10	6	1	29
Privacy and confidentiality	11	13	4	1	29
Determination of appropriate subject selection with regard to minors	11	9	6	1	27

*Some respondents did not answer some questions.

### Independence of ERCs

**Figure 2:**
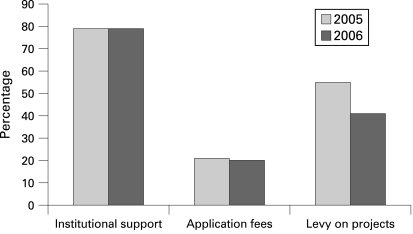
Sources of funds for 31 ethics review committees surveyed.

Membership of 10 committees was entirely by staff employed at the institution, while the rest had varying involvement of members from “outside” the parent institution, such as community members, local universities, religious organisations, non-governmental organisations, civic organisations and professional associations. A large proportion (77%) of the surveyed committees relied on funds received from the institutions where they were based in 2005 and 2006. Figure 2 shows that a relatively smaller proportion of respondents received levies on projects reviewed, whereas application fees were a source of funding for a much smaller number of committees, about 20% for both years.

## DISCUSSION

This survey covered 31 ERCs of 37 targeted institutions; 12 of the 31 committees also presented at workshops organised by AMANET. This is to our knowledge the first survey ever to cover so many countries and institutions; previous surveys in Africa have been on a smaller scale and arguably less comprehensive. Furthermore, this survey included anglophone (eight institutions), francophone (22 institutions) and lusophone (1) institutions. Although this survey received replies from most African regions, replies from central Africa were rare. The relatively high response rate could be attributed to the personal visits by the surveyors. Encouragingly, only two of the 37 institutions lacked ethics review committees and did not complete the questionnaire. This is a great improvement from previous times,^20^ when the absence of ethics committees in many African countries was common.

The World Health Organization publication *Operational guidelines for ethics committees that review biomedical research* (2000) states:

Countries, institutions, and communities should strive to develop ECs and ethical review systems that ensure the broadest possible coverage of protection for potential research participants and contribute to the highest attainable quality in the science and ethics of biomedical research. States should promote, as appropriate, the establishment of ECs at the national, institutional, and local levels that are independent, multi-disciplinary, multi-sectorial, and pluralistic in nature. ECs require administrative and financial support (p2).^21^

The survey shows that most institutions across sub-Saharan Africa have established ethics committees. However, in order to effectively review protocols, ERCs should be composed of members of diverse backgrounds; many ethics committees surveyed are not yet sufficiently multidisciplinary or multi-sectoral. There are also weaknesses relating to gender and age. The UNAIDS (Joint United Nations Programme on HIV/AIDS) guidelines stipulate that an ERC should have a minimum of five members, and there is no upper limit that is set by guidelines. The current study revealed that membership is still problematic for some ERCs in sub-Saharan Africa, with some having as few as three members and others 19 or more. The major reasons cited for the wide variation in membership include unwillingness of potential members to participate in the committees over and above their normal duties and the lack of compensation for the costs incurred in attending ERC meetings. These issues need to be addressed if ERCs are to function properly.

Independence of the committees from their institutions is influenced by a number of factors. First, a committee made up of members from the institution that hosts it, without external members, faces a high risk of bias in its work. Second, reliance on the parent institution for financial support also compromises the independence of the ERC. It is therefore imperative that the ultimate goal should be to enable ERCs to generate adequate operational funds in order to reduce financial reliance on host institutions and also to attract members from outside the parent institution. This is all the more important in sub-Saharan Africa, given the limited financial and skilled human resources available and very poor personnel remuneration. However, the cost of running an ERC needs to be determined, if cost-effective fees are to be charged to ensure self-sustainability. Although in developed countries such as the USA, efforts have been made to determine running costs,^22^ the authors are not aware of such attempts made elsewhere, especially in Africa.

Training of members before or upon joining an ERC would help to orient them in terms of the standard operating procedures in place and the ethical review procedures of the particular committee. Whereas the volume of trials being conducted in Africa is increasing, 92% of the surveyed committees reported that they are inadequately trained to properly review and monitor trials. Since it may not be feasible for committee members to take long leaves of absence to undergo long-term training away from their workplaces, workshops and web-based courses in health research ethics could go a long way towards meeting the training needs of the committees. Despite the increasing popularity of e-learning, only 4% (14/345) of the members surveyed have benefited from these opportunities, and this percentage may even decrease as committees become more independent, multi-sectoral and pluralistic. Reliance on traditional pedagogical methods, with all their drawbacks, particularly in the least-developed countries, may be the only opportunity.

The survey revealed the training needs of ethics committees in sub-Saharan Africa. A closer examination of the responses is guiding the development of ongoing training of members of ethics committees and will be invaluable in the development of upcoming training of investigators.

### Harmonisation of ethical review process

A study conducted in the USA showed variable decisions by different ERCs that reviewed the same protocol for a multi-centre genetic epidemiological study.^23^ Given the diversity of the ERCs in Africa, especially the weaknesses cited above, there is a high likelihood of diverse decisions if study protocols will be subjected to these committees, particularly in upcoming multi-centre studies. The need to harmonise ethical review in Africa is considered to be urgent. The harmonisation could first focus on procedural aspects of the ethical review process and subsequently address substantive aspects of ethical review, which could be more challenging than the former. However, for the harmonisation to be acceptable to the African ERCs and also effective in terms of improving the ethical review process, a participatory approach that includes all interested stakeholders is critical.

The survey also highlighted the need for clear roles and responsibilities of national ERCs in relation to institutional ERCs in countries where both national and institutional committees exist. The roles of the committees should be complementary rather than duplicative; it should be clear to the committees themselves and to potential applicants what type of health research protocols should be reviewed by the respective committees. Such clarity would go a long way towards minimising potential antagonism between the national and institutional ERCs of the same country.

## CONCLUSIONS

This paper provides useful public information on the status of ethics committees that stakeholders in biomedical research in Africa would find useful. The major constraints identified are shortage of resources and inadequate training of the ERC members. Sponsors of clinical trials in Africa will also find this a useful inventory when considering compliance of trial sites to international recommendations, and it is hoped that the ethical review process and oversight of research will always be taken into account at the design stage of research in order to include the activities in the budget and in the project time frame.

The gaps identified through this survey should be addressed through dedicated capacity-strengthening providing specifically identified and tailor-made support to ensure improvement, instead of conducting such surveys merely for academic purposes. A careful post-intervention survey using the same evaluation tools would be important to gauge the effectiveness of the interventions implemented, and the results should be widely disseminated for the benefit of the members of the scientific community who are involved in health research. Fostering collaborative efforts with other organisations involved in capacity-building of the ethical review process in Africa could go a long way towards minimising the risk of duplication of activities, which would waste resources.
